# Unusual Double Dislocation of Both Joints in a Same Finger: A Case Report

**DOI:** 10.5812/ircmj.9989

**Published:** 2013-11-05

**Authors:** Pankaj Kumar Mishra, Anuj Gupta, Suresh Chandra Gaur

**Affiliations:** 1 Orthopedics Department, Kanpur University, Allahabad, India

**Keywords:** IP Joints, MCP Joints, Dislocation, Finger

## Abstract

Simultaneous dislocation of both interphalangeal (IP) joints of same finger is a highly uncommon finding. And dislocation of metacarpophalangeal (MCP) joint along with interphalangeal joints of thumb are reported in literatures if, scarcely. Here we are reporting the three cases, comprising of double dislocation of IP joints in little finger in two patients and simultaneous MCP and IP joint dislocation in thumb in another third patients as a perusal of rare entity from the northern India.

## 1. Introduction

First case of double dislocation in a finger was documented in 1874 by Bartel (1). Dislocation of two joints in same finger is a rare event. Simultaneous dislocation of both interphalangeal (IP) joints of same finger is a highly uncommon finding. And dislocation of metacarpophalangeal (MCP) joint along with interphalangeal joints of thumb are reported in literatures if, scarcely. In various literatures till now, only sixty five cases of double dislocation of both IP joints in same finger and five cases of simultaneous MCP and IP joint dislocation in thumb have been previously reported.

## 2. Case Presentation

Here we are reporting the three cases (Table 1), comprising of double dislocation of IP joints in little finger in two patients and simultaneous MCP and IP joint dislocation in thumb in another third patient as a case of rare entity. All three cases were encountered between 12 years from 1999 to 2011 in M.L.N Medical College (city Allahabad) of INDIA. 

**Table 1.** General Data of Three Reported Cases

Sample no	Age, y	Sex	Diagnosis	Management
**1**	21	M	Dorsal dislocation of both I.P joints of right little finger	Closed reduction and splinting
**2**	28	M	Dorsal dislocation of both I.P joints of right little finger	Open reduction and K-wire fixation
**3**	25	M	Dorsal dislocation of MCP and IP joints of left thumb	Closed reduction and splinting

**Figure 1.**
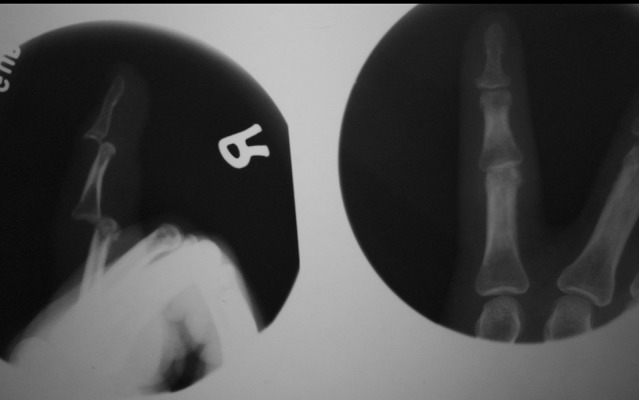
Showing Double Dislocation of IP Joints in Little Finger

**Figure 2.**
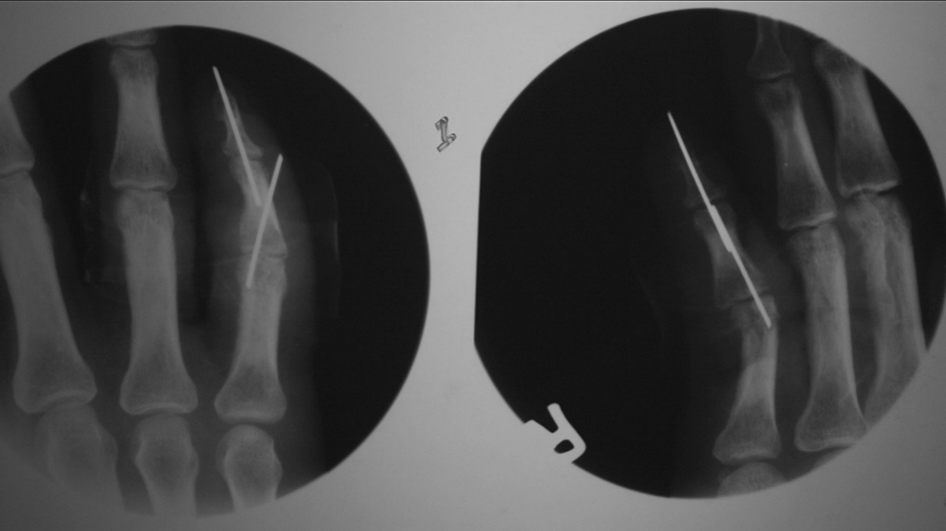
Open Reduction and Kirschner Wire Fixation of Both Joints

Our two cases of IP joints dislocation were adult male of 21 years and 28 years old. Younger one got injured (in June 1999) during cricket playing and reveled that mechanism of injury was hyperextention type. He presented to emergency department within two hours after injury. Radio graphically dorsal dislocation of both interphalangeal joints of little finger in dominant hand was confirmed. We used ring block anaesthesia and reduced by giving longitudinal traction and pressure over base of caudal phalanx. First done at distal and then at proximal interphalangeal joint and immobilized in intrinsic plus position for three weeks and then physiotherapy done. Second one presented (in January 2006) to us after seven days of trauma in his little finger. He was laborer by occupation, and injured during fall on the ground by keeping his right hand on the edge of wall for protecting himself. On radiography there was undisplaced condyler fracture of proximal phalanx along with dorsal dislocation of both interphalangeal joints. Close reduction tried for two times but failed then open reduction and Kirschner wire fixation done and immobilized (Figure 1 and 2). 

**Figure 3.**
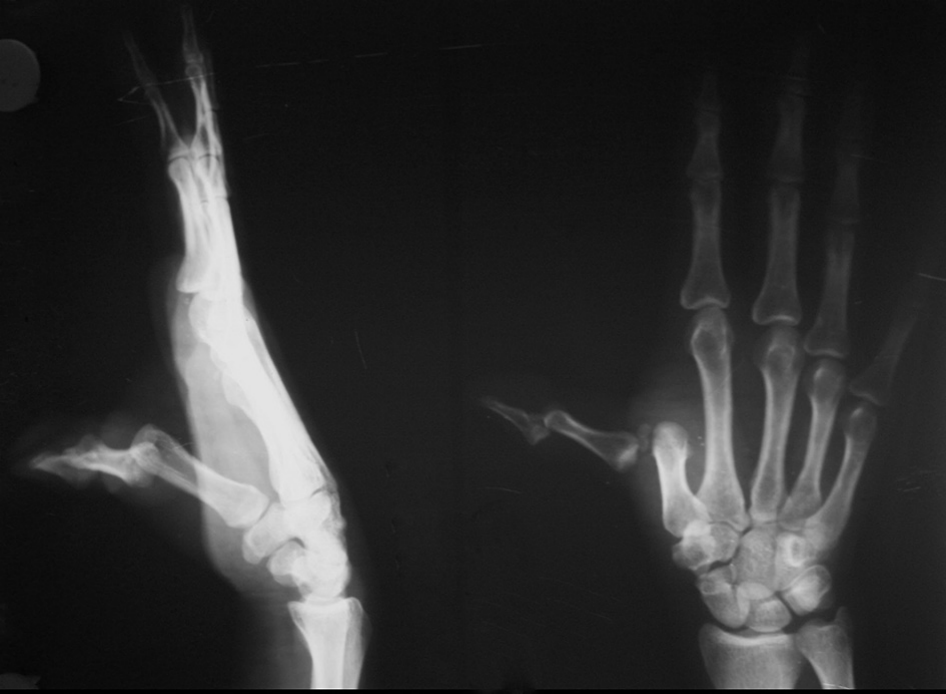
Showing Dislocation of MCP and IP Joints in Thumb

Our third case (in march 2011) of dislocation of MCP and IP joints of left thumb was present in twenty five year soccer player, who got injured during playing and visited to emergency department immediately. It was dorsal dislocation of both joints along with minor intraarticular fracture of base of distal phalanx (Figure 3). Reduction done in ring block anaesthesia, first IP joint reduced, then at MCP reduced. MCP joint got reduced by adducting to the metacarpal and hyperextending the joint, while proximal end of proximal phalanx is pushed against and over the metacarpal head with keeping flexion on IP joint to overcome action of flexor pollicis longus. Both joints splinted in 20-degree flexion for four week and then mobilized. 

After one year of trauma in all cases of IP joints dislocation, there was residual swelling in distal interphalangeal joint. Flexion movement was within normal limit at proximal and distal interphalangeal joints but extension lag of 15-degree was present at distal interphalangeal joints. In thumb dislocation the arc of motion was from 10-degree hyperextension to 60-degree flexion at IP joint and at MCP joint from zero degree to 30-degree flexion. All patients were gone through ethical consideration, and informed consent. Clinical variables were measured clinical and radiological.

## 3. Discussion

Review literature of Anderson et al. and study of Hutchison et al., and along with case report of Jahangiri SA et al. are the endorsements of rareness of this injury (2-4). In ten year Nakago et al. enumerated the sixteen cases of double dislocation of proximal and distal interphalangeal joints in same finger, while Edinburg hospital of hand surgery only reported eight cases of such type of injury in ten years, so these were published as a case report for the rarity (3, 5). As a rare case report, triple joints dislocations in a same finger also has been reported (6). Dorsal and lateral type of dislocation is most common type because of the nature of injury, which is hyperextension type and weak radial collateral ligament than ulnar ligament. Dislocation of finger occurs first at distal interphalangeal joints and if the magnitude of injury is more severe then, dislocation of proximal interphalangeal joints occurs. But the double dislocation in opposing direction, in same finger also has been reported (7). Mostly these injuries occur during sport activity like volleyball, baseball and football etc. Little finger followed by ring finger of dominant hand is most commonly involved. This is due to its weak ligaments and unsupported environment from surrounding (8-10). It may be associated with volar plate fracture of middle phalanx in distal interphalangeal dislocation and buttonholing through extensor aponeurosis by condyle of proximal phalanx in proximal interphalangeal joint dislocation, which may cause difficulty in reduction and entails open reduction. Double luxation of MCP and IP joints in thumb has been reported in very few number (11-16). Anteroposterior and true lateral radiograph are necessary to rule out possible appearance on radiograph and even clinically too until obscured by gross swelling. Reduction is obtained by applying longitudinal traction and pressure over dorsum of base of distal phalanx and, in the same way the proximal interphalangeal joint is reduced. Since it is quick but painful for short duration the reduction can be done even without the anesthesia. Local digital anaesthesia can also be used, because it allows more accurate clinical examination and ligament laxity is checked. In previously reported cases closed reduction have been achieved easily with or without the anaesthesia (17). In late presenting cases open reduction is needed but it compromises the result. Nasviaser et al. and Chan et al. have reported good results in their cases, but there are few reports, which ultimately needed fusion of joint due to pain (18, 19). For immobilization we avoided functional position because, in the deficiency of additional soft tissue stabilizer there is attendant loss of joint congruity, which can leads to secondary volar dislocation (20). Intrinsic plus position with 90 degree flexion at metacarpophalangeal joint and 15 degree flexion at interphalangeal joint was used for three week according to Kuczynski and Sprague recommendation followed by adequate physiotherapy.
